# Supplementary material for: BNT162b2 vaccine uptake and effectiveness in UK healthcare workers – a single centre cohort study
Source: Nat Commun. 2021 Jun 17;12:3698. doi: 10.1038/s41467-021-23927-x (PMC8211683; doi:10.1038/s41467-021-23927-x)
Supplement: Supplementary file 2 — Reporting Summary [file 41467_2021_23927_MOESM2_ESM.pdf]

nature research

Corresponding author(s): Tariq Azamgarhi

Last updated by author(s): May 19, 2021

Reporting Summary

Nature Research wishes to improve the reproducibility of the work that we publish. This form provides structure for consistency and transparency in reporting. For further information on Nature Research policies, see our Editorial Policies and the Editorial Policy Checklist.

Statistics

For all statistical analyses, confirm that the following items are present in the figure legend, table legend, main text, or Methods section.

n/a	Confirmed	
☐	☒	The exact sample size (n) for each experimental group/condition, given as a discrete number and unit of measurement
☐	☒	A statement on whether measurements were taken from distinct samples or whether the same sample was measured repeatedly
☐	☒	The statistical test(s) used AND whether they are one- or two-sided<br>*Only common tests should be described solely by name; describe more complex techniques in the Methods section.*
☐	☒	A description of all covariates tested
☒	☐	A description of any assumptions or corrections, such as tests of normality and adjustment for multiple comparisons
☐	☒	A full description of the statistical parameters including central tendency (e.g. means) or other basic estimates (e.g. regression coefficient) AND variation (e.g. standard deviation) or associated estimates of uncertainty (e.g. confidence intervals)
☐	☒	For null hypothesis testing, the test statistic (e.g. F, t, r) with confidence intervals, effect sizes, degrees of freedom and P value noted<br>*Give P values as exact values whenever suitable.*
☒	☐	For Bayesian analysis, information on the choice of priors and Markov chain Monte Carlo settings
☒	☐	For hierarchical and complex designs, identification of the appropriate level for tests and full reporting of outcomes
☐	☒	Estimates of effect sizes (e.g. Cohen's d, Pearson's r), indicating how they were calculated

Our web collection on statistics for biologists contains articles on many of the points above.

Software and code

Policy information about availability of computer code

Data collection	Microsoft Excel 2019
Data analysis	Statistical analysis was performed using SPSS version 25.0 (IBM, Chicago, ILs).

For manuscripts utilizing custom algorithms or software that are central to the research but not yet described in published literature, software must be made available to editors and reviewers. We strongly encourage code deposition in a community repository (e.g. GitHub). See the Nature Research guidelines for submitting code & software for further information.

Data

Policy information about availability of data

All manuscripts must include a data availability statement. This statement should provide the following information, where applicable:

- Accession codes, unique identifiers, or web links for publicly available datasets
- A list of figures that have associated raw data
- A description of any restrictions on data availability

The data that support the findings of this study are available from the corresponding author upon reasonable request.

Field-specific reporting

Life sciences study design

All studies must disclose on these points even when the disclosure is negative.

Sample size	2260 - We included all HCWs. The BNT162b2 efficacy was found to be high in phase 3 clinical trials. We estimated that early uptake of the COVID vaccine would be approximately 65% as this is the typical uptake we see with annual flu vaccinations at the study hospital. This would provide a vaccinated (circa 1470) and unvaccinated group (circa 800). We felt that the high community COVID prevalence rates (prevalence rates per 100,000 population were 1023.7 on 5th January) during the study period would give rise to sufficient infections to detect (either through assymptomatic screening or symptoms based testing) a difference in COVID infections rates between groups.
Data exclusions	1409 of 2260 healthcare workers received single dose BNT162b2 vaccine in our cohort study. We identified 25 healthcare workers who revived a single dose ChAdOx1 vaccine either before, during or after vaccinations. 25 was considered a small number and were excluded from the analyses because the main aim of the study was to assess the effectiveness of the BNT162b2 vaccine. 2 cases identifed during the follow up period were excluded because they had a positive test within 90 days of follow-up. This is stated in the manuscript.
Replication	Data were collected and checked by two of the authors. The statistician analyses were conducted in house and rechecked by an external statistician on 2 separate occasions.
Randomization	Healthcare worker were split into two groups depending on whether they received a single dose BNT162b2 vaccine. This was not random allocation. Two sets of Cox regression analyses were performed. Initially a simple 'unadjusted' comparison between partially vaccinated and unvaccinated groups was made. Subsequently, the groups were compared adjusting for demographic details found to vary significantly between groups. Hazard ratios were also adjusted for underlying COVID-19 infection rates in the London area. This was treated as a time-varying covariate, with different values for each day of the follow-up period.
Blinding	Only anonymised data were used in the analysis, therefore vaccination status of study participants was unknown to those collecting or analysing the data. Study participant anonymity also meant that the authors were unaware of HCWs intending to or not intending to take up the offer of a COVID vaccination, so they would not be influenced by that knowledge.

Reporting for specific materials, systems and methods

We require information from authors about some types of materials, experimental systems and methods used in many studies. Here, indicate whether each material, system or method listed is relevant to your study. If you are not sure if a list item applies to your research, read the appropriate section before selecting a response.

Materials & experimental systems

n/a	Involved in the study	
☒	☐	Antibodies
☒	☐	Eukaryotic cell lines
☒	☐	Palaeontology and archaeology
☒	☐	Animals and other organisms
☐	☒	Human research participants
☒	☐	Clinical data
☒	☐	Dual use research of concern

Methods

n/a	Involved in the study	
☒	☐	ChIP-seq
☒	☐	Flow cytometry
☒	☐	MRI-based neuroimaging

Human research participants

Policy information about studies involving human research participants

Population characteristics	age, gender, ethnicity and staff group
Recruitment	All staff were sent a COVID vaccination consent form by email. Staff without email access were offered a consent by their line manager face to face. Selection bias was avoided by offering COVID vaccination to all onsite healthcare workers at our hospital.
Ethics oversight	Our institutional review board is the Royal National Orthopaedic Hospital, Research and Innovation Centre. Anonymised data were collected and analysed so the need for consent was waived by the Royal National Orthopaedic Hospital, Research and Innovation Committee. Approval letter are attached to the resubmisssion for records.

Note that full information on the approval of the study protocol must also be provided in the manuscript.
